# Runoff mitigation via micro‐dams and conservation tillage—Numerical modeling of runoff and erosion from maize field trials

**DOI:** 10.1002/ieam.4546

**Published:** 2021-11-25

**Authors:** Stephan Sittig, Robin Sur, Dirk Baets

**Affiliations:** ^1^ knoell Germany GmbH Mannheim Germany; ^2^ Bayer AG Division Crop Science Monheim Germany; ^3^ Bayer AG Division Crop Science Machelen Belgium

**Keywords:** Conservation tillage, Mitigation, Modeling, Micro‐dams, Runoff

## Abstract

Runoff and erosion are the most important transport pathways of water, sediment, and associated pesticides from sloped agricultural fields. This results in the loss of fertile topsoil material, nutrients, irrigation water, and plant protection products (PPP) into adjacent surface water bodies. In the European and US risk assessment for the registration of PPP, runoff and erosion are numerically calculated with the simulation Pesticide Root Zone Model (PRZM) using the US Department of Agriculture (USDA) runoff curve number (CN) concept for the water movement and the MUSS equation to quantify the sediment transfer. This work presents an evaluation of maize field trials conducted in three seasons that considered micro‐dams (i.e., small earthen dams between the rows; also known as “furrow diking,” “furrow damming,” etc.) and/or conservation tillage (via subsoiling) as mitigation measures to investigate the effects on the reduction in runoff and erosion. Measured quantitative reductions and event‐wise calculated CN are presented. Furthermore, the trials were simulated using the PRZM over the complete vegetation period and runoff CN as well as parameter values of the MUSS erosion equation (a relative adaptation of the *C*‐factor) were inversely estimated. Compared with the control plots (i.e., conventional tillage), micro‐dams or conservation tillage reduced runoff by 24%–71% or 69%–89%, and erosion by 54%–81% or 91%–98%. Based on these data, a robust case can be made to lower CN or parameters in the MUSS equation for surface water exposure scenarios to consider the effects on predicted environmental concentrations (PECs) and estimated environmental concentrations (EECs). Mean resulting CN reductions by micro‐dams or conservation tillage were ascertained to be 6% (±2.5%) or 12% (±3.0%), the *C*‐factor was reduced by a factor of 0.1 (±0.15) or 0.48 (±0.19). Example calculations show reductions in the ranges of 11%–100% for PECs and 30%–98% for EECs. *Integr Environ Assess Manag* 2022;18:1348–1363. © 2021 Bayer AG Crop Science. *Integrated Environmental Assessment and Management* published by Wiley Periodicals LLC on behalf of Society of Environmental Toxicology & Chemistry (SETAC).

## Key Points

- The application of micro‐dams and conservation tillage can be adequately considered in risk assessment in the US and Europe.
- Calculating predicted environmental concentrations in the European (FOCUS) framework requires careful reassessment to adequately consider scenario specifics.
- Field trials can be simulated over complete seasons with the model PRZM with the aim to reproduce the total runoff water and soil erosion.
- The relative reduction of the parameter *C* of the MUSS erosion equation by micro‐dams and conservation tillage can be inferred from field trials over complete seasons.

## INTRODUCTION

In the European risk assessment framework for plant protection products (European Parliament and the Council of the European Union [EU], 2009), surface runoff from sloped agricultural fields is one building block for the estimation of risks for the aquatic environment, that is, surface water bodies adjacent to agricultural fields. The Forum for Co‐ordination of pesticide fate models and their Use (FOCUS; FOCUS, 2001, 2015) set the framework to assess predicted environmental concentrations (PECs) in standard water bodies for surface water (PECsw) and sediment (PECsed). For this process, the calculation of runoff water, erosion, and pesticide mass loadings to surface water bodies is conducted using the Pesticide Root Zone Model (PRZM; US Environmental Protection Agency [USEPA], 2006). In PRZM, water runoff and erosion are quantified with the US Department of Agriculture (USDA) Soil Conservation Service curve number (CN) methodology (US Department of Agriculture [USDA], 2004) and a watershed‐scale variation of the Universal Soil Loss Equation, that is, the MUSS equation (Williams, 1995), respectively. Based on the PRZM output, PECs are then calculated using the model TOXic substances in Surface Waters (TOXSWA; Beltman et al., 2014). There, additional entries via spray drift are accounted for to calculate maximum concentrations as well as time‐weighted averages. An analogous approach exists in US surface water exposure assessments with the PWC suite (US Environmental Protection Agency [USEPA], 2021), where PRZM is used in conjunction with the surface water body scenarios implemented in the variable volume water model (VVWM) to calculate estimated environmental concentrations for ecological risk assessments (EECs).

Mitigation becomes increasingly important to reduce the environmental impact of pesticides (Fox et al., 2021) and to comply with the objectives of the EU Green Deal (European Commission [EC], 2019) and the Farm‐to‐Fork strategy (European Commission [EC], 2020a). To prevent surface runoff and erosion, it is advised to minimize runoff generation both in‐field (via increasing infiltration) and at the edge‐of‐field (e.g., via vegetated filter strips [VFS]). However, measures that prevent runoff are much more effective than measures to stop already moving runoff (Alix et al., 2017).

One method is to create so‐called micro‐dams between the ridges of potato fields (Olivier et al., 2014) or in maize cultivation (Sui et al., 2016), also with the purpose of improving irrigation efficiency (Keshavarz et al., 2020). The required equipment is largely available commercially or available as advanced prototypes. An overview of the effects of quantitative reductions of runoff, erosion, and pesticide fluxes along with calculated effects on CN and resulting PECs is given in Sittig et al. (2020). Another method for runoff mitigation is conservation tillage. This term refers to a variety of practices, in contrast to conventional tillage. Gebhardt et al. (1985) group all tillage practices that reduce soil or water loss, when compared with mold‐board plowing, as conservation tillage. This results in little or no soil disturbance (Cueff et al., 2021). In their review paper, Alletto et al. (2010) characterize conservation tillage generally by the condition that more than 30% of the soil remains covered by crop residues after planting. The mitigating effect of conservation tillage on runoff and erosion needs further investigation (Du et al., 2021). Nevertheless, this practice not only prevents soil erosion but also compaction (Borrelli & Panagos, 2020; Gaynor et al., 1995) and reduces the emission of carbon dioxide (Reicosky, 1997).

In environmental risk assessment, either fixed default mitigation effectiveness values for specific measures can be used or a higher‐tier modeling can be conducted, for example, by deriving CNs for PRZM belonging to specific measures. A lowering of the CN by three points following the application of micro‐dams, other in‐field bunds, and reduced tillage was proposed by the SETAC MAgPIE workshop (Alix et al., 2017). More recent work generated additional robust data and demonstrated that this proposed reduction is too small (Sittig et al., 2020). The objective of the present work is to further enlarge the underlying database for the effect of micro‐dams and to add further data for conservation tillage as well as the combination of both strategies. The aim is to provide a better‐founded recommendation for the use in regulatory risk assessment and management.

In this study, in addition to quantitatively reporting the results of maize field trials, CN values and estimates for the *C*‐factor of the MUSS erosion equation (representing crop type and tillage method) have been derived. CN numbers were estimated both event‐wise, based on reported rainfall–runoff relationships and, alternatively, via simulating complete seasons with PRZM. By this means, we numerically quantified the effects of the dedicated mitigation measures micro‐dams and conservation tillage (with subsoiling, as defined in Alletto et al., 2010) in terms of reduction in runoff, and consequently, erosion and pesticide loadings. The field trial data allow both the abstraction and transfer to other conditions and a conservative and protective risk assessment because they were gained under very vulnerable conditions with large slopes (9%–16%) and large amounts of precipitation (191–337 mm). Example calculations within the European and the US framework for pesticide legislation were conducted to demonstrate the effect in the risk assessment.

## MATERIALS AND METHODS

The effects of the mitigation measures micro‐dams and conservation tillage in three maize field trials from 2018, 2019, and 2013 were assessed. Furthermore, these results were evaluated numerically to derive runoff CNs and parameter values for the *C*‐factor of the MUSS erosion equation. Example calculations demonstrate the impact of these measures on concentrations in surface water and sediment for the risk assessment with the European runoff scenarios (the PECs in surface water and sediment: PECsw and PECsed) and with the US scenario of Illinois corn (estimated environmental concentration: EECs).

For the trial from 2013, a PRZM simulation for the complete season is presented as an alternative to the event‐wise evaluation of micro‐dam application already presented for this trial in Sittig et al. (2020). The event‐wise evaluation is presented here again, this time with the conservation tillage practice and, for comparison, with the simulations of the complete season.

### Trials under consideration in this study

The field trials with maize cultivation (see Table 1 for details) were conducted on the Bayer “ForwardFarm” in Huldenberg, Belgium, in 2018, 2019, and 2013. The aim was to compare runoff and erosion under conventional tillage and conservation tillage with or without the installation of micro‐dams. After each runoff event, samples were taken for the quantification of the complete runoff water volume and erosion mass from each subplot.

**Table 1:** Details of the maize field trials under investigation, conducted on the Bayer ForwardFarm in Huldenberg (Belgium)

	Trial 2018	Trial 2019	Trial 2013
Devices	“ERuiStop” drum plow for micro‐dams, “Micheltand” for conservation tillage		Micro‐dams: disc plow, drum plow, “Micheltand” for conservation tillage
Soil type	Sandy loam (clay 15%, silt 74%)		Sandy loam (clay 15%, silt 74%)
Irrigation	No		No
Plot length (m)	24	18	24
Plot area (m^2^)	72	54	72
Slope (%)	9		9, 16

**Figure 1:**
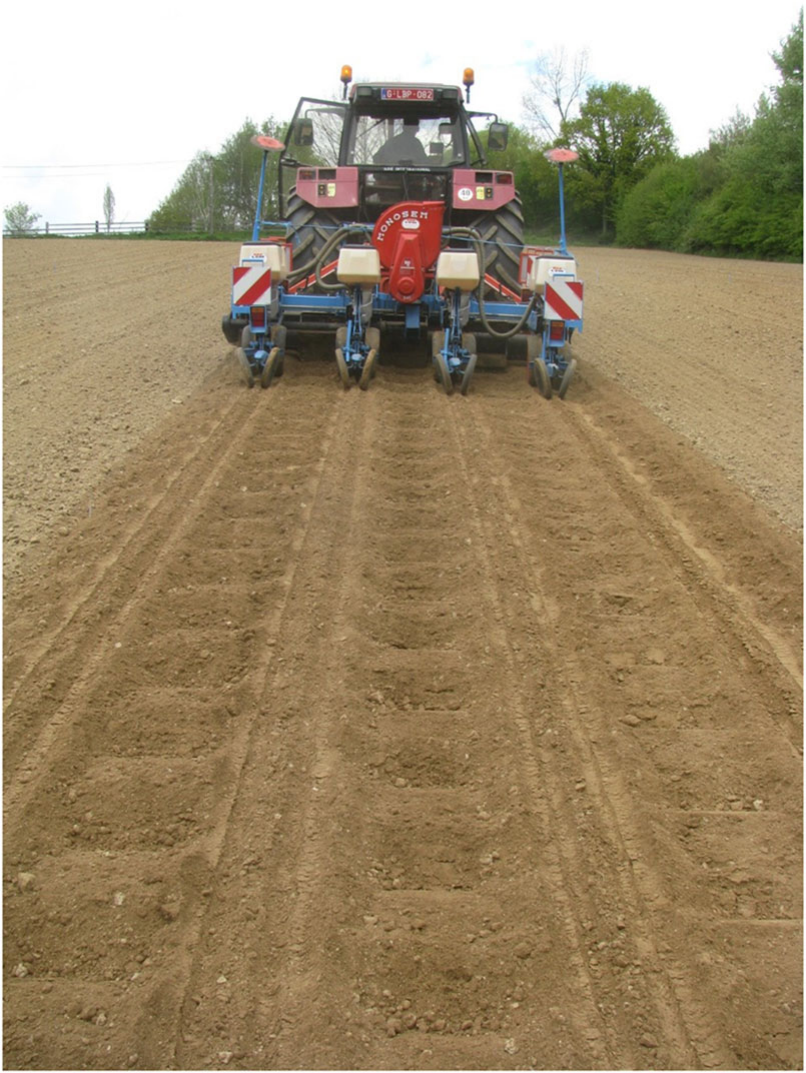
Resulting pattern of micro‐dams on the field (2018 and 2019 trials)

In the 2018 and 2019 trials, micro‐dams were applied to both conventionally tilled fields and to conservation tillage using the “ERuiStop” micro‐dam device from LSM (Jurbise, Belgium; Figure S1). The resulting pattern on the maize field is shown in Figure 1. The earthen micro‐dams had a rectangular shape of 40 times 20 cm, opposite to the slope, and a depth of 12 cm. They were spaced approximately 15 cm apart along the furrow. The “ERuiStop” anti‐erosion drum is a further development from the one used in the 2013 trial and is commercially available. In the 2013 trial, both a disc and a drum plow were applied to create micro‐dams of similar dimensions on conventionally tilled fields (images of the devices and the resulting patterns on the field are shown in Sittig et al., 2020).

**Figure 2:**
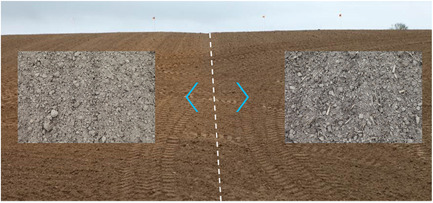
Historical field on the Bayer ForwardFarm in Huldenberg, undergoing conventional tillage (left) and conservation tillage (right) since 1997

Figure 2 shows the historical field that had been treated since 1997 with conventional tillage or conservation tillage on different subplots. In all trials reported here, the so‐called “Micheltand” from Steeno (Vichte, Belgium; Figure S2) was applied for conservation tillage. This device follows the principle of “subsoiling,” as defined in Alletto et al. (2010) in which the soil surface remains nearly covered with crop residues, and the soil is just lifted and the (sub)soil aerated. Hence, the whole surface is treated.

### Numerical evaluation of runoff and erosion

#### Theoretical background

The implementation of the USDA runoff CN concept (USDA, 2004) was conducted as described in Sittig et al. (2020). In short, the runoff CN relates precipitation events with corresponding runoff under the assumption of a certain fraction of precipitation that infiltrates immediately (Hortonian runoff).

Erosion is often quantified with the universal soil loss equation (USLE; Wischmeier & Smith, 1960). It was designed to calculate annual amounts of soil erosion and corresponding sediment yields from a watershed. The basis is a rainfall distribution. The application of the USLE to small plots and the calculation of event‐wise erosion results in large uncertainties. To overcome these, modified versions of the USLE were developed. For example, the MUSS equation (Williams, 1995) uses runoff amounts as direct input and is designed for small watersheds.

The model PRZM uses MUSS to quantify erosion (US Environmental Protection Agency [USEPA], 2014):

(1) $$X_{e}=0.79{\left(V_{r}q_{p}\right)}^{0.65}A^{0.009}K\;LS\;C\;P,$$

where *X*_e_ (t/day) is the event soil loss, *V*_r_ (mm) the volume of daily runoff event, *q*_p_ (mm/h) the peak storm runoff rate, *A* (ha) the field size, *K* (‐) the soil erodibility factor, *LS* (‐) the length‐slope factor, *C* (‐) the soil cover factor, and *P* (‐) the conservation practice factor.

Many of the factors in Equation (1) stem from other sources and are not taken from the field plots under consideration. The dimensionless factors *K*, *LS*, *C*, and *P* are listed in handbooks (Stone & Hilborn, 2015; US Environmental Protection Agency [USEPA], 1978) or defined in the corresponding FOCUS guidance documents (FOCUS, 2001, 2015), respectively.

The *C*‐factor is a combined measure that contains the influence of crop type and tillage method by multiplying these factors (Borrelli & Panagos, 2020). The crop type and tillage method used by default are related to a continuously fallow and tilled land. Hence, the objective is to derive relative numbers to compare different cropping and tillage systems (Stone & Hilborn, 2015). Panagos, Borrelli, Meusburger, Alewell, et al. (2015) present the combined effect that is considered for the estimation of the then called “management” *C*‐factor. They describe it as the product of the influences of the crop type, tillage, residue management, and soil cover. In this framework, as applied in the work herein, the effect of micro‐dams and/or conservation tillage were considered in the factor as being summarized in the concept of tillage.

#### Parameter estimation—Two alternative strategies

First, an event‐based calculation of the runoff CN was conducted as described in detail in Sittig et al. (2020). Briefly, the reported collected rainfall and the runoff water amounts were used to calculate the runoff CN. To arrive at a meaningful average value, the events were weighted with the corresponding amount of precipitation.

Second, the complete seasons were simulated with PRZM and values for CN and the *C*‐factor from the MUSS equation were derived. This approach has the advantage that environmental conditions such as weather, soil texture, and daily soil moisture are inherently considered.

Simulations with PRZM SW v4.3 in an automated calibration mode were conducted. The properties of the trial sites were parameterized in the PRZM input files (.inp; see Supporting Information). To this end, these files were set up following the PRZM manual (USEPA, 2006) with the appropriate MUSS parameters (except for *C*), according to the USLE fact sheet (Stone & Hilborn, 2015) to: *K* = 0.12 (sandy loam; OM > 2%); LS = 1.05 (trial of 2018), 0.90 (2019), 2.25 (2013: 16% slope), 1.05 (2013, 9%); *p* = 1. Additionally, the field sizes (e.g., 0.0054 ha), and slopes (9% or 16%, respectively) were required. The precipitation data were taken from stations near the trial sites for the 2018 and 2019 trials. In the study reports, rain amounts were given cumulatively (i.e., as they were gathered in the reservoirs) after an event of runoff and (if applicable) erosion. For the simulations, these reported amounts were adjusted with the information from the weather stations to better represent the actual temporal distribution of rain. The remaining meteorological data were obtained from the Meteorological Archival and Retrieval System (MARS), Grid No. 101094 (European Commission [EC], 2020b).

In a first step, the CN most suitable for the measured total annual runoff amounts were inferred. To this end, simulations with varying values for CN were conducted and the value fitting best was deducted. Subsequently, values for the MUSS *C*‐parameter were derived analogously to best match the measured annually eroded masses. Additionally, plots hitting the annual sums of runoff water and eroded sediment, together with their dynamics, were drawn.

### Example calculations of PECsw, PECsed, and EECs

The standard scenarios were adapted in terms of CN and MUSS *C*‐factor following the findings in this evaluation. For the European risk assessment, the simulations were conducted with the FOCUS standard scenarios R1 to R4 (FOCUS, 2001, 2015); the PECs considering mitigation were compared with the PECs from a corresponding regular assessment. For the US assessment, the Illinois corn scenario was taken.

Three substances were chosen for example calculations: FFA, TCM, and IMS, which represent substances of relative immobility (FFA: *K*_om_ = 125 L/kg; DT_50,water_ = 49.6 days, DT_50,soil_ = 18.3 days), intermediate mobility (TCM: *K*_om_ = 48 L/kg; DT_50,water_ = 26.1 days, DT_50,soil_ = 11.6 days), and high mobility (IMS: *K*_om_ = 19 L/kg; DT_50,water_ = 19.8 days, DT_50,soil_ = 2.7 days).

The concentrations of the European PECsw for the stream water body were evaluated as described below. The remaining values (i.e., PECsw for the pond scenario, PECsed, and EECs) were taken unchanged from the model outputs.

#### Procedure for PECsw stream scenarios

**Figure 3:**
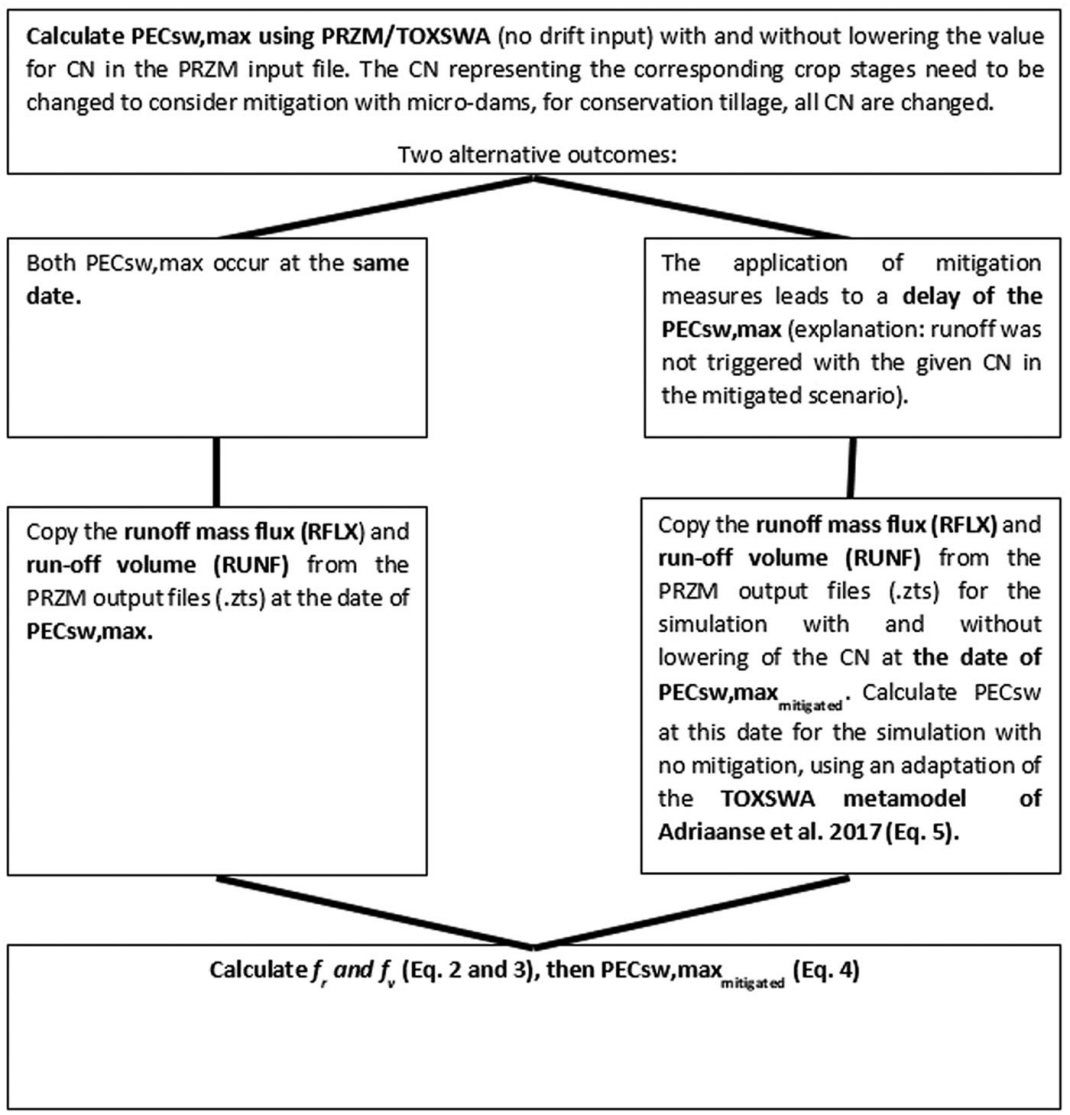
Procedure to calculate PECsw,max_mitigated_ for the FOCUS stream scenarios using FOCUS PRZM/TOXSWA after the application of micro‐dams and/or conservation tillage

The PECsw values according to FOCUS and calculated with the coupled models PRZM/TOXSWA need to be further processed if in‐field mitigation measures such as micro‐dams and conservation tillage in combination with a stream scenario are considered (in the pond scenario, no modification is required). In this scenario, a 1‐ha field is treated with a pesticide and equipped with a mitigation measure. The exposure endpoint is the concentration in surface water (and sediment) in an edge‐of‐field stream adjacent to this very field. Further dilution of the surface water is provided by baseflow from an upstream catchment that is only partially treated and equipped with the mitigation measure under consideration. Therefore, a post processing of the TOXSWA results must be conducted. Figure 3 shows the recalculation process, using Equations (2)–(5) below.

The procedure is based on the concept of the upstream catchment, in which only 20 ha of the 100‐ha large area receive mitigation measures (and pesticide application). This leads to a greater dilution of the edge‐of‐field PECsw as if the unprocessed output were directly considered (which would assume that the entire upstream catchment was mitigated as well). The strategy is in accordance with the handling of VFS as a mitigation measure in the context of the FOCUS landscape and mitigation framework (FOCUS Step 4; FOCUS, 2007), where only the treated field next to the water body and 20% of the upstream catchment have a VFS (ter Horst et al., 2009).

The fractional reduction in runoff (pesticide) mass flux (RFLX; from the PRZM output [g/cm^2^/day]) by the mitigation is calculated with:

(2) $$f_{r}=\frac{{\text{RFLX}}_{\text{no miti}}-{\text{RFLX}}_{\text{miti}}}{{\text{RFLX}}_{\text{no miti}}},$$

whereas the fractional reduction in runoff volume (RUNF; from the PRZM output [L/m^2^/day]) is given by:

(3) $$f_{v}=\frac{{\text{RUNF}}_{\text{no miti}}-{\text{RUNF}}_{\text{miti}}}{{\text{RUNF}}_{\text{no miti}}}.$$

The relationship between the mitigated and the unmitigated PECsw (mg/L) is defined by:

(4) $${\text{PEC}}_{\text{sw}}^{\text{mitigated}}={\text{PEC}}_{\text{sw}}^{\text{no mitigation}}\times \frac{1-f_{r}}{1-f_{a}fv},$$

where *f*_*a*_ (‐) is the fraction area treated = 0.208 (default FOCUS stream).

The calculation of ${\text{PEC}}_{\text{sw}}^{\text{no mitigation}}$ in an adaptation of Adriaanse et al. (2017) is conducted with:

(5) $${\text{PEC}}_{\text{sw}}^{\text{no mitigation}}=\frac{{\text{RFLX}}_{\text{no miti}}}{n}\times \frac{f_{a}}{\frac{\text{BASF}}{24}+\text{raindr}\times \frac{\text{INFLMON}}{24}+\frac{{\text{RUNF}}_{\text{no miti}}}{n}},$$

where *n* (‐) is the number of hours per day with simulated runoff (for calculation, the total precipitation per day must be divided in *n* 2‐mm steps); BASF (L/m^2^/day) the baseflow; default = 0.2 (approximate median of FOCUS R stream scenarios); raindr (‐) the fraction of INFL going into base flow (TOXSWA input); user‐defined: default = 0.1 (FOCUS definition); INFLMON (L/m^2^/day) the monthly average of INFL for the corresponding month (from PRZM output).

This procedure constitutes a refinement of the one presented in Sittig et al. (2020), in which the fractional reductions of runoff mass flux *f*_r_ and the runoff volume *f*_v_ were assumed to be identical. It is demonstrated later that those two variables have similar values.

Example calculation: The global maximum PEC for the R1 scenario (TOXSWA output) is 2.72 µg/L, occurring on 7 May 1984. Reducing the CN in the PRZM input leads to a global maximum on 20 May 1984 of 1.34 µg/L. This PEC was calculated under the intrinsic consideration of reduced runoff via micro‐dams in the complete scenario, including the entire upstream catchment. Therefore, the correction is required to assume only mitigation on the proportion of the upstream catchment that receives pesticide application in compliance with the defined scenario conditions.

The runoff fluxes or runoff volumes in the regular or modified scenario are 1.3E–2 and 4.6E–5 mg/m^2^/day and 1.6 and 0.2 L/m^2^/day, respectively. This leads to values for the fractions *f*_r_ and *f*_v_ of 0.997 and 0.89. Using Equation (5), a PECsw for the standard scenario on 20 May 1984, with the precipitation on that day being 19.9 mm (i.e., *n* = 10) and the monthly average of INFL in May 1984 of 1.3 L/m^2^/day, a PECsw of 1.61 is calculated. Inserting this into Equation (4) leads to a PEC with mitigation of 6.82E–3 µg/L.

## RESULTS AND DISCUSSION

### Effects of micro‐dam application and conservation tillage on the measured quantities and the calculated runoff CNs in the event‐based evaluation

Table 2 lists the effects of micro‐dams and conservation tillage. The measures led to a decrease in runoff from the fields, which is reflected in lower runoff CNs (here: calculated for each event). Consequently, following the reduced runoff water amounts, the eroded sediment quantities were reduced. Table S1 (2018 and 2019 trials) and Table S2 and Table S3 (trial of 2013) in the Supporting Information provide the corresponding experimental data, together with the event‐wise derived runoff CN. Table S4 and Table S5 in the Supporting Information list the erosion amounts for the 2018, 2019, and 2013 trials, respectively.

**Table 2:** Reductions of runoff volumes, erosion quantities, and derived runoff curve numbers (CN; means, calculated event‐wise) after the application of micro‐dams (MD) and/or conservation tillage (CsT) compared with conventional tillage (CvT)

	CvT			CsT			Red. CsT versus CvT	Red. MD + CsT versus CvT	Red. MD + CsT versus MD + CvT	Red. CsT versus MD + CvT
	No MD	MD	Red. (%)	No MD	MD	Red. (%)	(%)	(%)	(%)	(%)
2018										
Runoff (mm)	7.3	4.2	43	1.2	0.7	46	83	91	83	71
CN	75	72	4.0	67	66	1.9	11	13	8	7
Erosion (kg/ha)	2371	1046	56	62	34	43	97	99	97	94
2019										
Runoff (mm)	9.7	3.3	66	3.0	1.5	49	69	84	55	9
CN	73	70	4.4	66	65	1.9	10	12	7	6
Erosion (kg/ha)	6655	1203	82	599	204	66	91	97	83	50
2013 (slope 16%)										
Runoff (mm)	7.3	5.5/2.3^a^	24/68^a^	0.78	NA	NA	89	NA	NA	86/66^a^
CN	69	68/63^a^	1.6/8.6	63	NA	NA	8.6	NA	NA	5/0^a^
Erosion (kg/ha)	1030	480/260^a^	54/75	17	NA	NA	98	NA	NA	96/93^a^
2013 (slope 9%)										
Runoff (mm)	4.8	2.5/1.4^a^	47/71^a^	0.62	NA	NA	87	NA	NA	75/56^a^
CN	67	66/64^a^	2.1/3.9^a^	63	NA	NA	5.9	NA	NA	3/1^a^
Erosion (kg/ha)	420	170/80^a^	59/81^a^	17	NA	NA	96	NA	NA	90/79^a^
Mean reductions	CN points	%								
MD + CvT versus CvT (*n* = 6)	3 (±1.7)	4.0 (±2.4)								
MD + CsT versus CsT (*n* = 2)	1 (±0.0)	1.5 (±0.0)								
CsT versus CvT (*n* = 4)	6 (±1.5)	8.7 (±1.7)								
MD + CsT versus CvT (*n* = 2)	9 (±0.5)	11.5 (±0.5)								
MD + CsT versus MD + CvT (*n* = 2)	6 (±0.5)	7.7 (±0.6)								
CsT versus MD + CvT (*n* = 6)	3 (±1.9)	4.4 (±2.7)								

*Notes*: Mean reductions are calculated over all corresponding experimental setups where applicable, standard deviations are given in brackets.

^a^ For disc and drum plow micro‐dam creating technology, respectively.

Abbreviations: NA, not applicable; Red., reduced.

Generally, conservation tillage led to larger decreases in both runoff and erosion than micro‐dams: The overall mean reductions (and standard deviations) by micro‐dams versus conservation tillage were 53% (±17%) versus 82% (±17%) for runoff and 68% (±12%) versus 96% (±3%) for erosion.

#### Quantitative runoff

In the 2018 trial, in one of the runoff events (on 24 May), the reservoir for the collection of runoff had overflown; this event was not considered in the further evaluations for both runoff and erosion. On another date, runoff was observed from the control plot only (0.24 mm on 30 August)—both micro‐dams and conservation tillage completely suppressed runoff generation. On other occasions, micro‐dams or conservation tillage also completely stopped runoff.

On 14 August 2018, a collected rainfall event of 26 mm led to a runoff event of 4.38 mm (in the control plot), whereas on 2 May 2018, 34 mm of rain led to 0.58 mm runoff only. Due to the evaluation methods, these divergences in the observations were not considered here, because both the general condition of the micro‐dams and differences in rain intensities could be the reason, and neither aspect was considered in this assessment.

In the 2019 trial, the first five reported rain events (with a collected rain amount up to 20 mm on 6 May) did not initiate runoff generation. This was presumably because of the relatively new condition of the micro‐dams because, for example, a rainfall of only 11 mm on 3 September led to a runoff of 0.57 mm on the control plot.

Generally, over all trials, the application of micro‐dams reduced the amount of runoff considerably—between 24% (2013 trial, using the disc plow, 16% slope) and 71% (2013 trial, using the drum plow, 9% slope) in the conventional tillage, and approximately 50% in the conservation tillage in the 2018 and 2019 trials. Furthermore, a considerable decrease in efficiency during the season cannot be noted. From field observations, the authors assumed a seasonal degradation of approximately 20%.

Conservation tillage in general reduced the runoff by 69%–89%. Comparing the two “extremes” in the 2018 and 2019 trials, that is, conventional tillage with no micro‐dams with conservation tillage with micro‐dams, led to a decrease of 91% and 84%, respectively.

#### Calculated runoff CNs

Under conventional tillage, the application of micro‐dams resulted in an average decrease over all studies in the CN by 1.6%–8.6%, that is, 1–6 points from starting points in the range 67–75. The reduction under conservation tillage was one point in both 2018 and 2019, in addition to the general reduction caused by the conservation tillage of eight (2018) or seven (2019) points. Comparing the two “extremes” in 2018 and 2019, that is, conventional tillage with no micro‐dams with conservation tillage with micro‐dams revealed a decrease of 13% and 12%, respectively. This equals a lowering of nine and eight points from 75 and 73, respectively. In the 2013 trial, conservation tillage alone led to a decrease in the CN of six and four points from 69 and 67 for the slope of 16% and 9%, respectively.

The overall mean reduction resulting from the application of micro‐dams with conventional tillage of three points is well in line with the results from Sittig et al. (2020; note that the event‐wise evaluation of the 2013 trial was already part of this previous study).

In the 2018 trial, reductions resulting from micro‐dams of up to five points were derived on single events throughout the season, that is, the micro‐dams were also “functioning” later in the season. For example, for the event of 8 September, the installation of micro‐dams led to a reduction from 85 to 80, the same quantitative effect of five points as on 2 May: from 67 to 62. Conservation tillage added up to five points (2 May) to the reduction, with the average being one point. In 2019, two events with reductions of six points stand out from the rest, whereas on one occasion, the CN of 73 was not reduced—in the very small event of 19 August with only 0.06 mm of runoff in the control plot. In the 2013 trial, the drum plow (creating micro‐dams) generally was more effective than the disc plow—in the setup with 15%–16% slope it was even more effective than conservation tillage.

#### Erosion masses

The application of micro‐dams approximately halved the masses that were eroded in the 2018 trial, in both conventional and conservation tillage. The event of 14 August was by far the most extensive, resulting in almost 2.2 t/ha eroded mass, which was reduced to 1.0 t/ha with micro‐dams. In conservation tillage, only the very small amount of 26 and 32 t/ha with and without micro‐dams was eroded during this event.

In 2019, micro‐dams led to an even larger decrease of 82% and 66%, respectively. The practice of conservation tillage reduced the amount of eroded masses by more than 90% over conventional tillage. As with the 2018 trial, one event far exceeded all of the others: on 11 June 3.2 t/ha was eroded and diminished to 0.5 t/ha by micro‐dams. Generally, in the 2019 trial, almost three times more mass was eroded than in 2018.

In the 2013 trial, micro‐dams reduced the eroded masses between 54% and 81%. With conservation tillage, almost no erosion was observed. Throughout the season, less mass was eroded than in the 2018 and 2019 trials—even in the subtrial with a slope of 15%–16%.

### Simulations of the complete seasons using PRZM

Table 3 shows the results of the simulations of runoff and erosion with PRZM regarding the complete seasons. In general, the quantitative reproduction of both runoff and erosion could be achieved quite well. The CN lie in the expected range (with the FOCUS value for R1, R3, and R4 being 82; for R2 being 78) and demonstrate a good consistency between the different trials. It must be noted that those CN were chosen that lead at least to the amount of measured runoff. In each case, the *C*‐factor was adjusted to perfectly fit the observed erosion masses (see the Supporting Information for an example input file for the estimation of the *C*‐factor).

**Table 3:** Simulations of the maize field studies over whole seasons with the model PRZM: resulting runoff curve numbers (CN) and erosion *C*‐factors

	CN	*C‐*factor	*C*‐factor rel. to CvT	Erosion		Runoff	
	(‐)	(‐)	(‐)	Sim. (t/ha)	Meas. (t/ha)	Sim. (mm)	Meas. (mm)
**2018**							
**CvT**	80	7.17	1.00	2.37	2.37	8.10	7.30
**MD** **+** **CvT**	75	7.14	1.00	1.05	1.05	4.60	4.15
**CsT**	66	2.33	0.32	0.06	0.06	1.40	1.22
**MD** **+** **CsT**	64	2.03	0.28	0.03	0.03	0.80	0.66
**2019**							
**CvT**	74	16.2	1.00	6.70	6.70	10.60	9.70
**MD** **+** **CvT**	67	13.8	0.85	1.20	1.20	3.90	3.30
**CsT**	66	9.10	0.56	0.60	0.60	3.20	3.00
**MD** **+** **CsT**	63	7.00	0.43	0.20	0.20	1.70	1.54
**2013**							
**Series A (slope 16%)**							
**CvT**	81	0.74	1.00	1.04	1.03	7.41	7.28
**Disc plow (MD)**	80	0.45	0.61	0.49	0.48	6.17	5.51
**Drum plow (MD)**	76	0.79	1.07	0.26	0.26	2.76	2.30
**CsT**	72	0.27	0.36	0.02	0.02	0.91	0.78
**Series B (slope 9%)**							
**CvT**	79	1.23	1.00	0.42	0.42	5.15	4.78
**Disc plow (MD)**	76	1.20	0.98	0.17	0.17	2.76	2.54
**Drum plow (MD)**	74	1.12	0.91	0.08	0.08	1.67	1.38
**CsT**	71	1.00	0.81	0.02	0.02	0.64	0.62
**Mean Reductions**	**CN points**	**%**	***C*** **‐factor rel. reduction**				
**MD** **+** **CvT versus CvT (** ***n*** **=** **6)**	4 (±1.9)	6 (±2.5)	0.10 (±0.15)				
**MD** **+** **CsT versus CsT (** ***n*** **=** **2)**	3 (±0.5)	4 (±0.8)	0.09 (±0.04)				
**CvT versus CsT (** ***n*** **=** **4)**	10 (±2.5)	12 (±3.0)	0.48 (±0.19)				
**MD** **+** **CsT versus CvT (** ***n*** **=** **2)**	14 (±2.5)	17 (±2.6)	0.64 (±0.07)				
**MD** **+** **CsT versus MD** **+** **CvT (** ***n*** **=** **2)**	8 (±3.5)	10(±4.3)	0.57 (±0.15)				
**MD** **+** **CvT versus CsT (** ***n*** **=** **6)**	5 (±2.8)	6.6 (±3.5)	0.36 (±0.24)				

*Notes*: Results are given for conventional tillage (CvT) and conservation tillage (CsT), respectively; both disc and drum plow were used to prepare micro‐dams (MD) in the trial of 2013; mean reductions are calculated over all corresponding setups, standard deviations are given brackets.

Abbreviations: CsT, conservation tillage; CvT, conventional tillage; MD, micro‐dams; Meas., measured; Sim., simulated.

Curve numbers in the range of 63 (CsT with micro‐dams in 2019) and 81 (conventional tillage [CvT] in 2013, 16% slope) were found. To fit the measured quantities of eroded masses, a value for the *C*‐factor between 0.27 (CsT in 2013, 16% slope) and 16.2 (CvT in 2019) was inferred. A decrease in the CN resulting from the mitigation measures did not automatically coincide with lower values for the MUSS *C*‐factor, as can be seen for example in the trial of 2013. There, the application of micro‐dams with CvT by using the drum plow led to a decrease in the CN of five points, whereas the *C*‐factor increased here from 0.74 to 0.79. As the runoff volume is a variable in the MUSS equation, the strong reduction in runoff had to be compensated by an increase in the *C*‐factor to match the observed sediment reduction.

The numbers of both observed runoff and erosion events were considerably greater than those resulting from the simulations. For example, in the experiment of 2019 with CvT and no micro‐dams, 10 events of runoff and erosion were observed, whereas in the PRZM simulations, only six events resulted. The single simulated events were mostly of higher quantity than the measured ones to compensate for this. Generally, the focus of this simulation study was on the reproduction of annual sums—single events can hardly be expected to be reflected in the PRZM simulations. It must be noted that the MUSS equation is not well documented and is not intended to be a realistic mechanistic simulation of the erosion process. The objective is a quantification in the regulatory process.

Because the intensity of rainfall cannot be considered because of the nature of the reported data and the daily resolution in the simulations (in PRZM the total daily amount of rainfall is automatically subdivided in 2‐mm segments for each hour of the day), a relatively small reported total amount of precipitation could be associated with an intensive runoff and erosion event. The likely high intensity of such an event is not represented in a simulation. This was presumably the case, for example, in the 14 August 2018 trial, where 26 mm of rain led to a runoff event from the conventional tilled field with no micro‐dams of 4.4 mm and an exceptional amount of eroded mass of 2193 t/ha. On the other hand, 34 mm of rain on 2 May only led to 0.58 mm of runoff and 78 t/ha of eroded mass.

**Figure 4:**
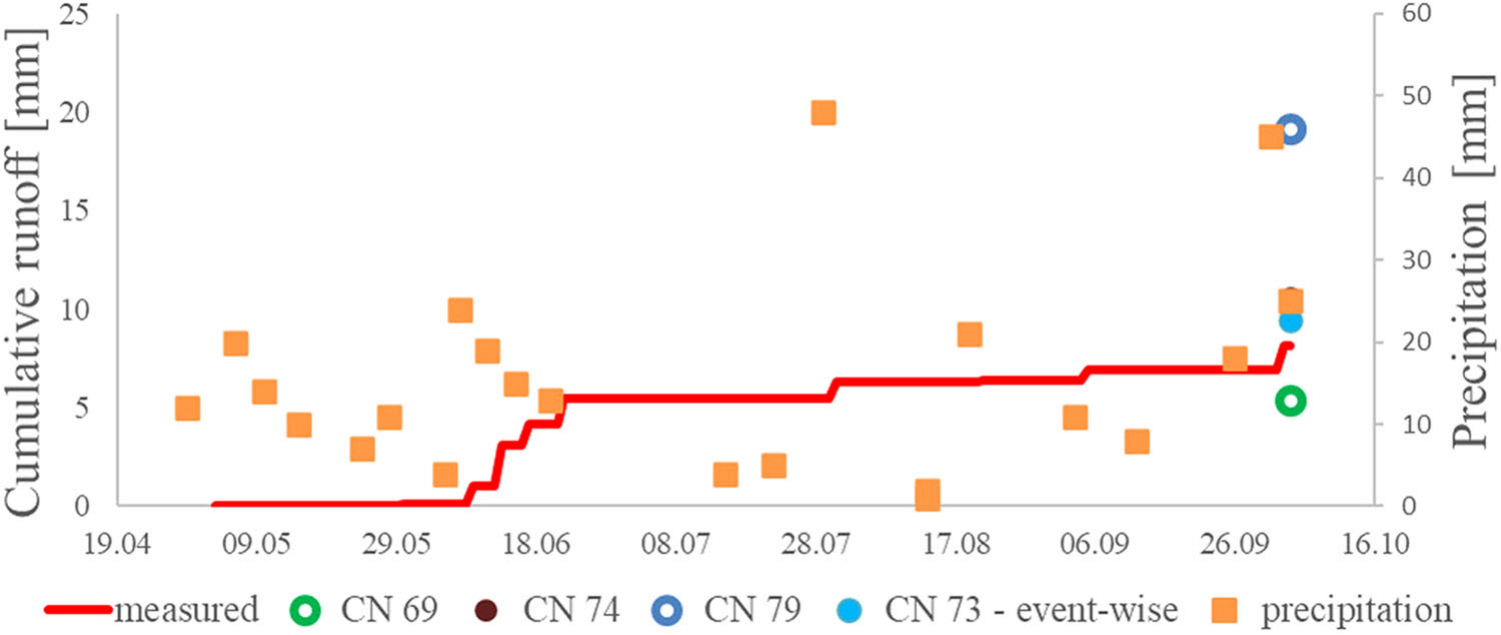
Measured cumulative runoff amounts in the 2019 trial for the control setup with conventional tillage (no micro‐dams), together with the corresponding simulated runoffs by PRZM with a runoff curve number (CN) of 74 (drawn behind last precipitation) and, for comparison, simulations with CNs of 79 and 69. The amount for the CN of 73 stems from the event‐wise evaluation. Precipitation was collected event‐wise

**Figure 5:**
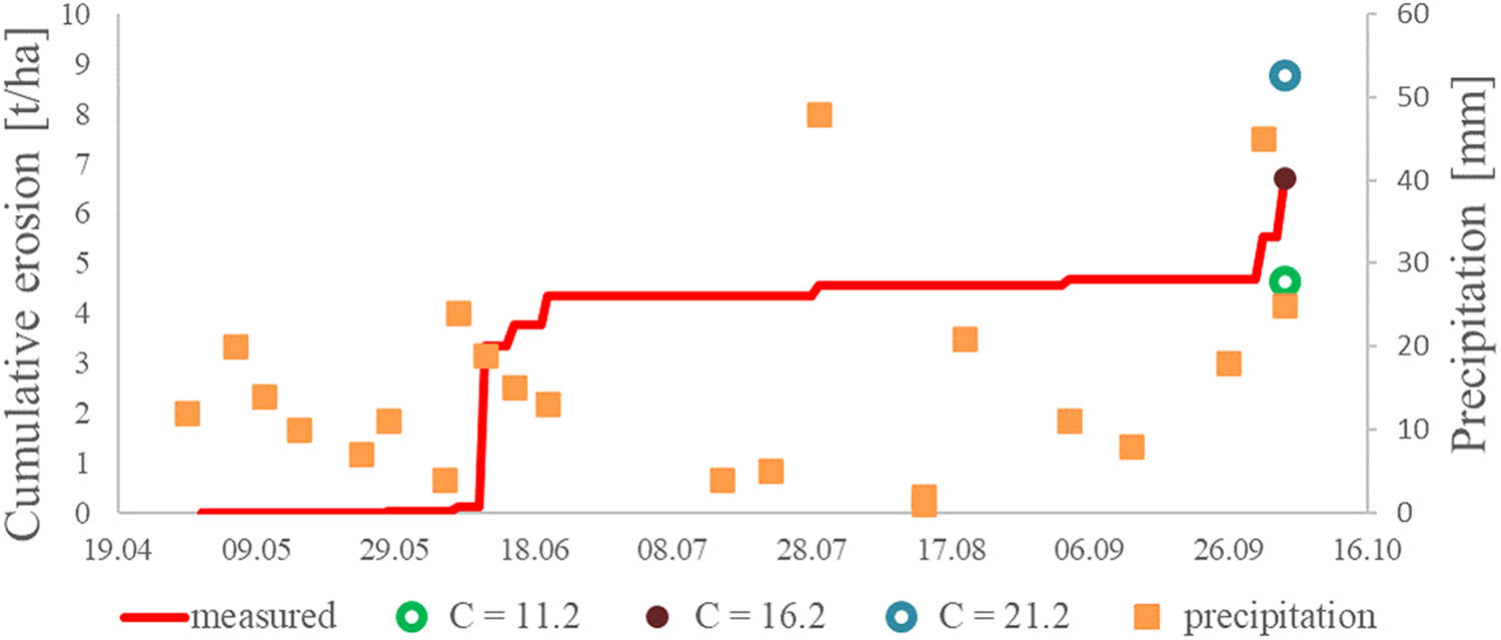
Measured cumulative erosion amounts in the 2019 trial for the control setup with conventional tillage (no micro‐dams), the corresponding simulated erosion masses by PRZM (MUSS *C*‐factor 16.2), and, for comparison, simulations with MUSS *C*‐factor 21.2 and 11.2. Precipitation was collected event‐wise

Figure 4 exemplarily depicts the measured and simulated runoff amounts for the control setup from the 2019 trial (i.e., conventional tillage, no micro‐dams). For this setup, a CN of 74 was found suitable via calibration. To demonstrate the sensitivity of the value for the CN, resulting runoff for the CN of 69 and 79 are depicted as well. These calculations resulted in a runoff of 21.8 mm for a CN of 79 and 5.8 mm for a value of 69, compared with 10.5 mm with a CN of 74. As an example, the outcome of the event‐wise evaluation is depicted as well, that is, a mean, precipitation‐weighted CN of 73. In this case, event‐wise evaluation and simulation led to an almost identical CN. Generally, the accordance between both evaluation strategies was quite good (see chapter 3.4 and Table 6). Figure 5 shows the associated eroded masses. For the quantification of erosion, a *C*‐factor of 16.2 (equals 6.7 t/ha) resulted from the calibration. The resulting masses for a *C*‐factor of 11.2 (4.6 t/ha) and 21.2 (8.3 t/ha) are shown for comparison. In both figures, the precipitation amounts collected after the single events are drawn. Five consecutive rain events at the beginning of June led to the first runoff and erosion amounts detected in the reservoirs. During summer, only a few more events of runoff and erosion were observed, before (at the end of the experimental period) two events led to considerable amounts of runoff and erosion at the beginning of October.

### Example calculations of PECs for surface water and sediment

In Table 4, results of example calculations using FOCUS PRZM and FOCUS TOXSWA are listed to demonstrate the effect of the herein derived reductions of CN and *C*‐factors. Analogous results for the US risk assessment (EECs) are presented in Table 5.

**Table 4:** Example calculations of predicted environmental concentrations for surface water (PECsw) and sediment (PECsed) for three example substances (FFA, TCM, and IMS)

	PECsw (µg/L)					PECsed (µg/kg)				
	CvT	MD + CvT	Red. (%)	CsT	Red. (%)	CvT	MD + CvT	Red. (%)	CsT	Red. (%)
**FFA (215 g/ha)**										
**R1 pond**	0.090	0.025	72	3.5E − 03	96	0.22	0.063	72	9.0E − 03	96
**R1 stream**	2.7	6.8E − 03	100	0.037	99	0.61	0.35	44	0.10	83
**R2 stream**	1.8	0.34	81	0.14	93	0.51	0.25	51	0.14	72
**R3 stream**	0.88	0.028	97	1.1E − 03	100	0.25	0.040	84	5.3E − 03	98
**R4 stream**	7.2	3.1	58	0.58	92	2.4	2.1	12	1.3	46
**TCM (30 g/ha)**										
**R1 pond**	5.2E − 03	1.0E − 03	80	9.5E − 05	98	8.2E − 03	1.6E − 03	80	1.8E − 04	98
**R1 stream**	0.41	0.030	93	2.9E − 03	99	0.073	0.014	81	4.3E − 03	94
**R2 stream**	0.31	0.057	81	2.5E − 03	99	0.061	0.032	48	4.0E − 03	93
**R3 stream**	0.033	6.2E − 04	98	2.2E − 06	100	6.1E − 03	8.6E − 04	86	8.0E − 06	100
**R4 stream**	1.0	0.43	58	0.081	92	0.24	0.22	11	0.14	45
**IMS (1.56 g/ha)**										
**R1 pond**	5.8E − 05	6.0E − 06	90	<1.0E − 6	100	1.0E − 04	1.0E − 05	90	1.0E − 06	99
**R1 stream**	9.6E − 03	1.8E − 04	98	1.8E − 05	100	1.6E − 03	9.0E − 05	94	2.8E − 05	98
**R2 stream**	3.3E − 03	6.1E − 04	81	<1.0E − 6	100	6.5E − 04	3.4E − 04	47	<1.0E − 6	100
**R3 stream**	8.7E − 05	1.3E − 06	99	<1.0E − 6	100	1.6E − 05	2.0E − 06	88	<1.0E − 6	100
**R4 stream**	0.026	0.011	58	2.0E − 03	92	5.7E − 03	5.1E − 03	11	3.2E − 03	44
**Mean reductions (%**)										
**PECsw**										
**MD** **+** **CvT**	83									
**CsT**	97									
**PECsed**									
**MD** **+** **CvT**	60									
**CsT**	84									

*Notes*: 14 days before emergence; conducted within the European framework using FOCUS PRZM and FOCUS TOXSWA applying the herein derived effects on parametrization; results for conventional tillage (CvT), conventional tillage with micro‐dams (CvT MD), and conservation tillage (CsT).

**Table 5:** Example calculations of estimated environmental concentrations for ecological risk assessments (EECs; 1‐in‐10 year conc.) for three example substances (FFA, TCM, and IMS; at the date of emergence) conducted within the US risk assessment framework (Scenario: Illinois corn)

	EEC (ppb)			Reduction (%)	
	CvT	MD + CvT	CsT	MD + CvT	CsT
**FFA (240 g/ha)**					
**Water column**					
**Pond 1‐day average**	1.71	1.15	0.67	33	61
**Reservoir 1‐day average**	3.68	2.58	1.57	30	57
**Benthic**—**total/dry sed.**					
**Pond 1‐day average**	8.9	5.10	2.63	43	71
**Reservoir 1‐day average**	34.3	17.88	6.60	48	81
**TCM (30 g/ha)**					
**Water column**					
**Pond 1‐day average**	0.19	0.12	0.071	37	63
**Reservoir 1‐day average**	0.44	0.29	0.17	34	61
**Benthic**—**total/dry sed.**					
**Pond 1‐day average**	0.37	0.2	0.096	46	74
**Reservoir 1‐day average**	1.27	0.58	0.25	54	80
**IMS (1.56 g/ha)**					
**Water column**					
**Pond 1‐day average**	2.90E − 03	1.50E − 03	7.10E − 05	48	98
**Reservoir 1‐day average**	6.80E − 04	3.50E − 04	1.70E − 04	49	75
**Benthic**—**total/dry sed.**					
**Pond 1‐day average**	2.10E − 03	9.40E − 04	4.90E − 04	55	77
**Reservoir 1‐day average**	7.30E − 03	2.60E − 03	1.10E − 03	64	85
**Mean reductions** (**%**)	**MD** **+** **CvT**	**CsT**			
**Water column**					
**Pond 1‐day average**	39	74			
**Reservoir 1‐day average**	38	65			
**Benthic**—**total/dry sed.**					
**Pond 1‐day average**	48	74			
**Reservoir 1‐day average**	56	82			

Abbreviations: Cst, conservation tillage; CvT, conventional tillage; MD, micro‐dams.

Following the findings noted in Table 3, the CNs were reduced by 6% and the *C*‐factor was reduced by a factor of 0.1 following the application of micro‐dams and by 12% and 0.48 for conservation tillage.

With conservation tillage, the PECsw were reduced drastically: for all three substances by 92%–100%, that is, by 97%, on average. Micro‐dams on CvT led to a mean reduction of 83%. Generally, the reductions of PECsed were smaller than for PECsw. This is probably because, in the scenario definition, the sediment load comes from the treated agricultural field only and not from the upstream catchment. Consequently, the dilution from the untreated area has a smaller influence.

Comparing the procedure conducted in Sittig et al. (2020) using the example of TCM R1 with micro‐dams reveals an *f*_r_ of 0.69 and an *f*_v_ 0.68. Consequently, assuming the fraction of runoff water (*f*_v_) only (Equation 4) leads to virtually the same PECsw. This confirms findings for VFS that the fractional reduction in runoff water flow is a very good predictor for the associated pesticide reduction in runoff (Reichenberger et al., 2019).

The EECs were reduced with micro‐dams by 30%–49% in the water column and by 30%–64% in the benthic. The corresponding reductions by conservation tillage were 57%–98% and 71%–85%. The degree of reduction generally increases with mobility, that is, IMS > TCM > FFA.

**Figure 6:**
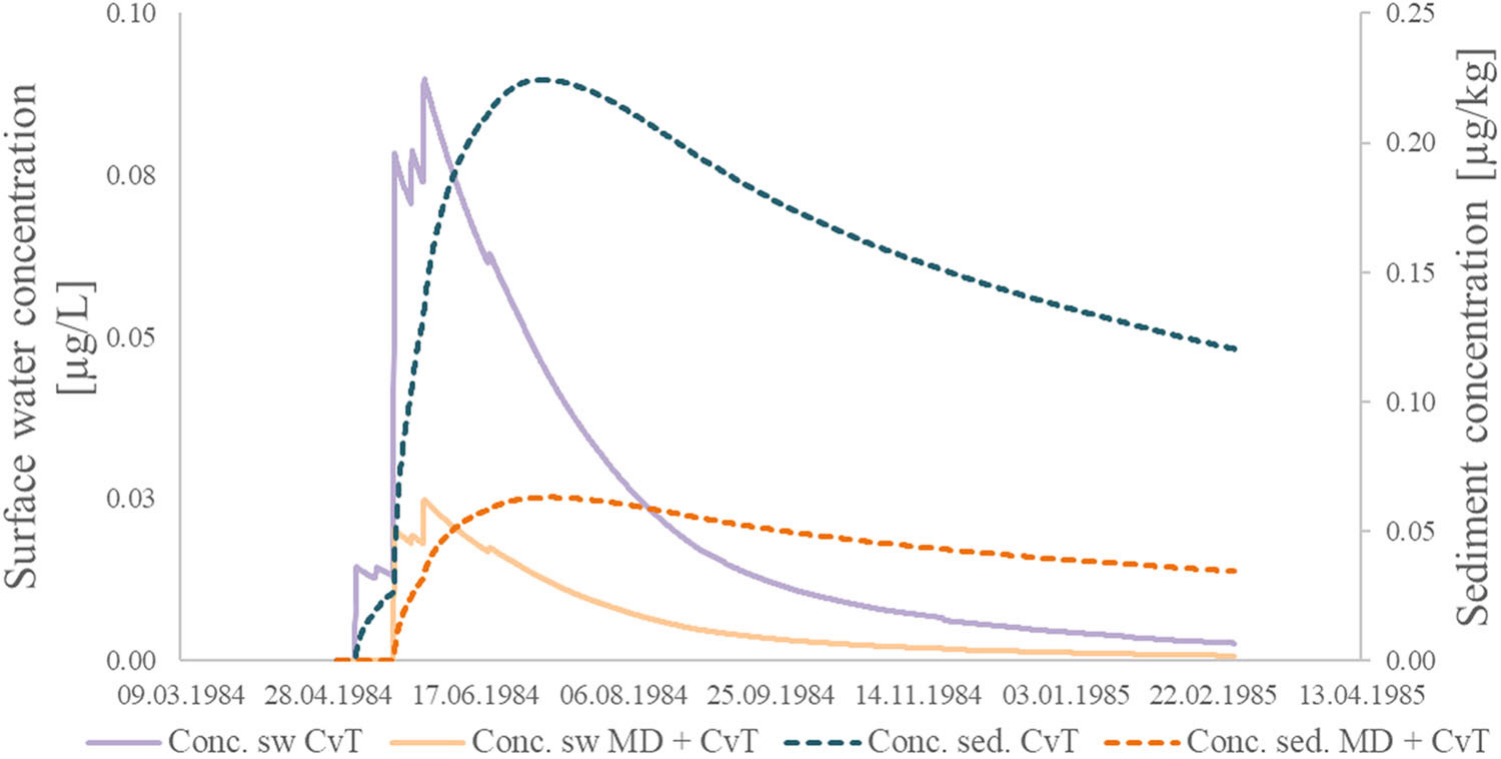
Concentration dynamics in the surface water (sw; solid lines) and in the sediment (sed.; dashed lines): example calculations using FOCUS PRZM and FOCUS TOXSWA for the substance FFA and the FOCUS standard scenario R1 pond, considering conventional tillage (CvT) with and without micro‐dams (MD)

The mitigation measures affect both the timing and the amplitude of environmental concentrations. For elucidation of the effects, the temporal dynamics of surface water and sediment concentrations with and without the application of micro‐dams are depicted in Figure 6. In this example with the application of FFA and the R1 pond scenario, the maximum concentration is reduced from 0.090 to 0.025 µg/L in the surface water and from 0.22 to 0.063 µg/kg in the sediment. The timing of the substance's arrival in the surface water is shifted by 13 days from 7 May to 20 May. Furthermore, the tailing of the concentration curves, that is, the background concentrations throughout the year, are considerably reduced after the application of micro‐dams.

### Comparison of the runoff CN evaluation: event‐wise versus PRZM simulations

The event‐based evaluation reflects the single relationship between precipitation and runoff. The PRZM simulation, on the contrary, contains a complete season—albeit not designed to mechanistically simulate the exact process of each single event, an overall annual quantification of both runoff and erosion is achieved. Furthermore, PRZM is the model used in risk assessment, applying the CN concept with one value for almost the entire cropping period. Consequently, quantifying the complete runoff process over the cropping period reflects the risk assessment procedure appropriately.

On the other hand, a calibration with the goal of representing the complete sum of the yearly runoff does reflect the actual measured runoff in the events only to a limited extent—single events with a large amount of runoff determine the resulting parameter estimation more than smaller events do. Therefore, a side‐by‐side comparison of both approaches leads to a useful complementary assessment of this complex situation and can be applied to evaluate the robustness of the final result. A close agreement in the outcome of both procedures would certainly indicate a robust parameter estimate; however, in case of a discrepancy, the results from the better of both approaches (simulation) ought to be used, as discussed before.

Table 6 lists a comparison of the two evaluation strategies in terms of the CN. Aside from a few exceptions, the CN from the simulations are higher than the event‐based ones. This is probably because a higher CN is required to initiate runoff in the simulations. To meet the measured overall sums, fewer events with larger runoff amounts resulted. Overall, the resulting reduction in the CN score is very similar: three or four points (4% or 6%, respectively) for the impact of micro‐dams following the event‐based evaluation and the simulation, respectively.

**Table 6:** Comparison of the runoff curve numbers (CNs) resulting from event‐based evaluations or simulations over the complete season with PRZM; results are given for the setups under conventional tillage (CvT) or conservation tillage (CsT), respectively, both with and without micro‐dams (MD)

	2018		2019		2013 (16%)		2013 (9%)	
	Event‐based	Simulation	Event‐based	Simulation	Event‐based	Simulation	Event‐based	Simulation
**CvT**	75	80	73	74	69	81	67	79
**MD** **+** **CvT**	72	75	70	67	–	–	–	–
**Disc plow (MD)**	–	–	–	–	68	80	66	76
**Drum plow (MD)**	–	–	–	–	63	76	64	74
**CsT**	67	66	66	66	63	72	63	71
**MD** **+** **CsT**	66	64	65	63	–	–	–	–
**Respective mean CN reductions**								
**Event‐based and simulation**								
**MD**	**Points**	**%**						
**CvT**—**event‐based**	3 (±1.7)	4 (±2.4)						
**CvT**—**simulation**	4 (±1.9)	6 (±2.5)						
**CsT**—**event‐based**	1 (±0.0)	2 (±0.0)						
**CsT**—**simulation**	3 (±0.5)	4 (±0.8)						
**CsT**								
**event‐based**	6 (±1.5)	9 (±1.7)						
**simulation**	10 (±2.5)	12 (±3.0)						
**MD** **+** **CsT**								
**event‐based**	9 (±0.5)	11 (±0.5)						
**simulation**	14 (±2.5)	17 (±2.6)						
**MD** **+** **CsT versus MD** **+** **CvT**								
**event‐based**	6 (±0.5)	8 (±0.6)						
**simulation**	8 (±3.5)	10 (±4.3)						
**CsT versus MD** **+** **CvT**								
**event‐based**	3 (±1.9)	(±2.7)						
**simulation**	5 (±2.8)	7 (±3.5)						

Note that the derivation of parameters describing the erosion was conducted based on PRZM simulations only (Table 3).

### General discussion

#### Erosion description with the MUSS equation—considerations on the multiplicative factors *C* and *P*

In this study, values for the *C*‐factor of the MUSS were derived. In the scientific literature, the distinction between the *C*‐factor and the *P*‐factor is not always clearly defined. Both factors quantify the effects of dedicated management practices to reduce soil loss. Panagos, Borrelli, Meusburger, Alewell, et al. (2015) define the *C*‐factor as cover management factor, which can be most readily influenced by the farmers via management practices such as conservation tillage. In Panagos, Borrelli, Meusburger, van der Zanden, et al. (2015) the *P*‐factor is the quantification of support practices such as contour farming, strip cropping, terracing, and subsurface drainage.

As stated in USEPA (1978), the product of the factors except *C* and *P* in the USLE defines conditions with continuously clean‐tilled fallow. They describe the value for *C* to be influenced by many variables—besides crop and weather, management practices such as mulching, tillage, land use, and crop residuals and their interactions are named. Furthermore, they also assign the factor *P* to supporting cropland practices such as contour tillage, strip cropping, and terrace systems.

The objective of this study was not to derive exact values for the parameters of the MUSS equation, but to provide the relative reduction stemming from the mitigation measures.

#### Further considerations of (subsoil) conservation tillage

Derivations of CN belonging to seasonal German crop conditions were conducted in Auerswald and Haider (1996), who demonstrate the decline of the value for the CN depending on the soil cover by the plants. Before that, runoff CN reduction following the practice of conservation tillage was exhaustively investigated in Rawls et al. (1980), focusing on the amount of residues left on the field and fraction of the field covered by residues, respectively. The effect of conservation tillage in the form of no‐till planting on the runoff CN was investigated in Volkmer (2014). Studies further investigating the effects of conservation tillage practices on runoff and erosion are deemed necessary (Du et al., 2021), especially in the context of subsoiling, as was done in this study with the Micheltand device.

To the authors' knowledge, the results presented herein constitute the first evaluation of the effects of subsoiling on runoff CN and MUSS *C*‐factor. Future investigations can be conducted to further enhance the database in this aspect. Seifert et al. (2001) reported no decrease in water runoff after subsoil tillage. This is in sharp contrast to the findings presented here. On the other hand, Du et al. (2021) found the presence of crop cover was mostly correlated with both runoff and erosion reductions among a variety of conservation management practices.

## SUMMARY AND CONCLUSIONS

An evaluation of three field trials with maize cultivation is presented. The fields were treated with different tillage strategies: conventional tillage, micro‐dams between the rows, and conservation tillage (using a subsoiler). In addition to reporting the measured reductions of runoff water and erosive soil transport, two alternative ways to describe the results numerically are presented. These first calculate runoff CNs based on the reported rainfall–runoff relationship for the single events and, second, simulations over the whole seasons. The latter were conducted with the model PRZM to estimate values for both the CN and the *C*‐factor of the equation to describe erosion in the runoff model PRZM (i.e., the MUSS equation).

The amount of runoff water was reduced considering micro‐dams in combination with conventional tillage or conservation tillage by only 24%–71% or 69%–89%, respectively. The corresponding sediment loads dropped by 54%–81% or 91%–98%. The evaluations result in average runoff CN reductions after the application of micro‐dams with CvT of three and four points (4% or 6%), following the event‐based or the simulation of complete seasons, respectively. The application of conservation tillage resulted in a reduction of six and 10 points (9% and 12%, respectively). The reduction in erosion can be quantified by the *C*‐factor with a relative decrease of 10% and 48%, for micro‐dam application or conservation tillage, respectively.

It is recommended to adapt the procedure to consider risk mitigation measures in regulatory modeling by the findings given in this study, that is, the installation of micro‐dams in maize cultivation indicates a modified input, for example, a lowering of the CN in the simulations with FOCUS PRZM by four points, based on the simulations of complete seasons. Analogously, the *C*‐factor could be reduced by a factor of 0.1. Under conservation tillage, a CN reduction of 10 points and a reduction in the *C*‐factor of 0.48 is indicated, whereas this procedure is not limited to maize cultivation, but seems appropriate to other crops (e.g., cereals and sugar beet) as well. The corresponding PECs in the surface water stream scenarios can subsequently be calculated using the procedure described here—those in the sediment and the pond scenarios can be gained from the corresponding output directly. Example calculations show reductions in the ranges of 11%–100% for PECs (58%–100% for PECsw and 11%–100% for PECsed) and 30%–98% for EECs (30%–98% in water and 43%–85% in sediment).

## CONFLICT OF INTEREST

The authors declare that there are no conflicts of interest.

## Supporting information

This article includes online‐only Supporting Information.

The Supplementary information file includes photographs from the field trials, further experimental data, and an example of the model input files for the PRZM model.

## Data Availability

The underlying data are available in the Supplementary Information file.
