# Supplementary material for: Runoff mitigation via micro‐dams and conservation tillage—Numerical modeling of runoff and erosion from maize field trials
Source: Integr Environ Assess Manag. 2021 Nov 25;18(5):1348–63. doi: 10.1002/ieam.4546 (PMC9546288; doi:10.1002/ieam.4546)
Supplement: Supplementary file 1 — The Supplementary information file includes photographs from the field trials, further experimental data, and an example of the model input files for the PRZM model. [file IEAM-18-1348-s001.docx]

Supplementary Information


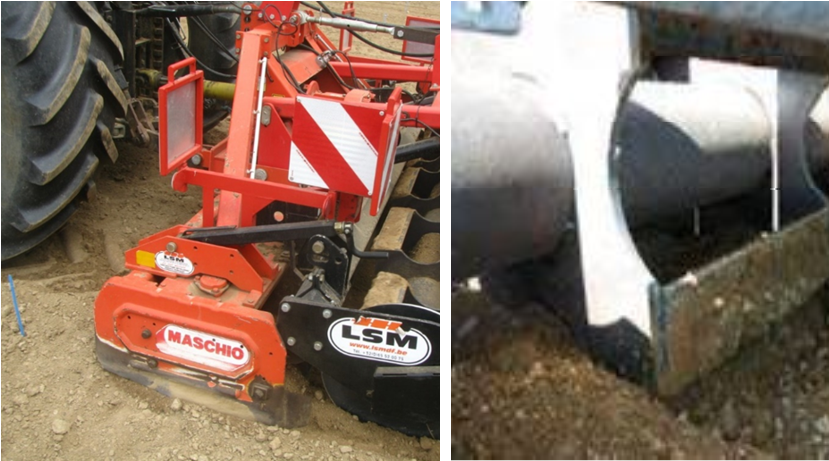


Figure S1  “ERuiStop” - device for the installation of micro-dams from LSM (Trials of 2018 and 2019).


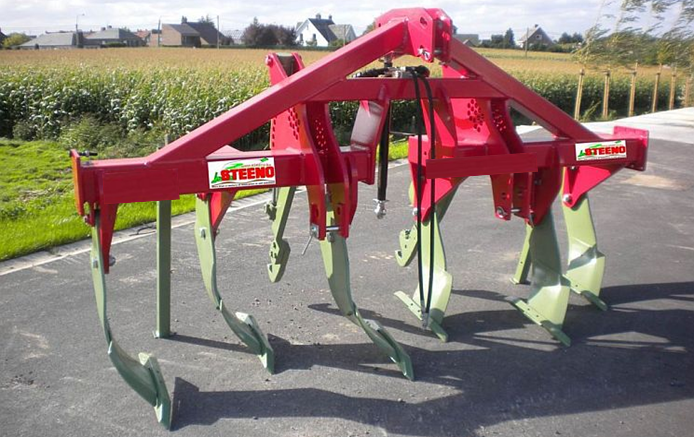


Figure S2 “Micheltand”: device to conduct conservation tillage.

## Experimental data

### Runoff amounts and calculated curve numbers

Table S1: Results of the field trials in 2018 and 2019 on the Bayer “ForwardFarm” in Huldenberg: precipitation (P), runoff amounts, and the resulting calculated curve numbers (CN; means and means normalized to the amount of P); the trials were conducted with conventional tillage (CvT) or conservation tillage (CsT), with or without micro-dams (MD)

| Date | P [mm] | Runoff [mm] | | | | Curve number [-] | | | | | |
| --- | --- | --- | --- | --- | --- | --- | --- | --- | --- | --- | --- |
|  |  | CvT | | CsT | | CvT | | Red. | CsT | | Red. |
|  |  | No MD | MD | No MD | MD | No MD | MD |  | No MD | MD |  |
| 2018 trial | | | | | | | | | | | |
| **02 May** | 34 | 0.58 | 0.06 | 0.46 | 0.03 | 67 | 62 | 5 | 66 | 61 | 5 |
| **15 May** | 2 | no runoff | | | | - | - | - | - | - | - |
| **24 May** | 39 | overflow | | | | - | - | - | - | - | - |
| **04 Jun** | 24 | 0.06 | 0.01 | 0.02 | 0.01 | 70 | 69 | 1 | 69 | 69 | 0 |
| **08 Jun** | 4 | no runoff | | | | - | - | - | - | - | - |
| **05 Jul** | 4 | no runoff | | | | - | - | - | - | - | - |
| **10 Aug** | 16 | 0.35 | 0.14 | 0.06 | 0.05 | 82 | 80 | 2 | 78 | 78 | 0 |
| **14 Aug** | 26 | 4.38 | 3.61 | 0.47 | 0.49 | 84 | 83 | 1 | 73 | 73 | 0 |
| **17 Aug** | 14 | 0.39 | 0.17 | 0.06 | 0.01 | 84 | 82 | 2 | 81 | 80 | 1 |
| **28 Aug** | 8 | no runoff | | | | - | - | - | - | - | - |
| **30 Aug** | 9 | 0.24 | 0.00 | no runoff | | 89 | - | 4 | - | - | - |
| **08 Sep** | 16 | 1.01 | 0.13 | 0.10 | 0.04 | 85 | 80 | 6 | 79 | 78 | 1 |
| **24 Sep** | 34 | 0.30 | 0.04 | 0.06 | 0.03 | 65 | 62 | 3 | 62 | 61 | 1 |
| **mean** |  | 0.91 | 0.52 | 0.15 | 0.08 | 78 | 74 | 4 | 73 | 72 | 1 |
| **mean,**  **norm. to P** |  |  |  |  |  | 75 | 72 | 3 | 67 | 66 | 1 |
| 2019 trial | | | | | | | | | | | |
| **29 Apr** | 12 | no runoff | | | | - | - | - | - | - | - |
| **06 May** | 20 | no runoff | | | | - | - | - | - | - | - |
| **10 May** | 14 | no runoff | | | | - | - | - | - | - | - |
| **15 May** | 10 | no runoff | | | | - | - | - | - | - | - |
| **24 May** | 7 | no runoff | | | | - | - | - | - | - | - |
| **28 May** | 11 | 0.11 | 0.00 | no runoff | | 85 | - | - | - | - | - |
| **05 Jun** | 4 | no runoff | | | | - | - | - | - | - | - |
| **07 Jun** | 24 | 0.91 | 0.33 | 0.2 | 0.1 | 77 | 74 | 3 | 72 | 72 | 0 |
| **11 Jun** | 19 | 2.04 | 0.65 | 0.5 | 0.2 | 86 | 80 | 5 | 80 | 77 | 3 |
| **15 Jun** | 15 | 1.13 | 0.24 | 0.3 | 0.1 | 87 | 82 | 5 | 83 | 80 | 2 |
| **20 Jun** | 13 | 1.26 | 0.33 | 0.4 | 0.2 | 89 | 85 | 4 | 86 | 84 | 1 |
| **10 Jul** | 0 | no runoff | | | | - | - | - | - | - | - |
| **15 Jul** | 4 | no runoff | | | | - | - | - | - | - | - |
| **22 Jul** | 5 | no runoff | | | | - | - | - | - | - | - |
| **29 Jul** | 48 | 0.85 | 0.63 | 0.5 | 0.4 | 59 | 58 | 1 | 57 | 57 | 1 |
| **12 Aug** | 1 | no runoff | | | | - | - | - | - | - | - |
| **13 Aug** | 2 | no runoff | | | | - | - | - | - | - | - |
| **19 Aug** | 21 | 0.06 | 0.04 | no runoff | | 73 | 73 | 0 | - | - | - |
| **03 Sep** | 11 | 0.57 | 0.39 | 0.1 | 0.0 | 89 | 88 | 1 | 85 | 82 | 3 |
| **12 Sep** | 8 | no runoff | | | | - | - | - | - | - | - |
| **26 Sep** | 18 | no runoff | | | | - | - | - | - | - | - |
| **01 Oct** | 45 | 1.24 | 0.39 | 0.5 | 0.3 | 62 | 58 | 4 | 59 | 58 | 1 |
| **04 Oct** | 25 | 1.52 | 0.31 | 0.5 | 0.1 | 78 | 72 | 6 | 74 | 71 | 3 |
| **mean** |  | 0.97 | 0.33 | 0.37 | 0.19 | 79 | 74 | 4 | 79 | 78 | 2 |
| **mean,**  **norm. to P** |  |  |  |  |  | 73 | 70 | 3 | 66 | 65 | 1 |

Table S2: Results of the field trial in 2013 on the Bayer ForwardFarm in Huldenberg; precipitation (P) and runoff amounts for the 3 distinct management practices: conventional tillage (CvT), disc or drum plough to create micro-dams, and conservation tillage (CsT)

| Date | P [mm] | Runoff measured [mm] | | | | | | | |
| --- | --- | --- | --- | --- | --- | --- | --- | --- | --- |
|  |  | Repetition A (slope 15–16%) | | | | Repetition B (slope 8–9%) | | | |
|  |  | CvT | disc | drum | CsT | CvT | disc | drum | CsT |
| **22 May** | 24 | 0.14 | 0.31 | 0.17 | 0.14 | 0.15 | 0.18 | 0.09 | 0.10 |
| **30 May** | 38 | 1.65 | 0.37 | 0.49 | 0.35 | 1.70 | 0.40 | 0.25 | 0.25 |
| **25 Jun** | 20 | 0.50 | 0.08 | 0.18 | 0.07 | 0.58 | 0.08 | 0.05 | 0.06 |
| **02 Jul** | 20 | 0.08 | 0.03 | 0.04 | 0.03 | 0.17 | 0.03 | 0.04 | 0.02 |
| **30 Jul** | 36 | 2.68 | 2.85 | 0.72 | 0.08 | 1.25 | 1.11 | 0.51 | 0.05 |
| **12 Aug** | 27 | 2.01 | 1.74 | 0.63 | 0.05 | 0.72 | 0.69 | 0.38 | 0.06 |
| **11 Aug** | 28 | 0.08 | 0.04 | 0.00 | 0.00 | 0.06 | 0.00 | 0.00 | 0.00 |
| **20 Sep** | 32 | 0.02 | 0.02 | 0.02 | 0.01 | 0.04 | 0.03 | 0.02 | 0.03 |
| **17 Oct** | 43 | 0.11 | 0.07 | 0.06 | 0.06 | 0.11 | 0.02 | 0.04 | 0.05 |
| **Total** | 268 | 7.28 | 5.51 | 2.30 | 0.78 | 4.78 | 2.54 | 1.38 | 0.62 |

Table S3: Results of the field trial in 2013 on the Bayer ForwardFarm in Huldenberg; estimated curve numbers (CN) for the 3 distinct management practices: conventional tillage (CvT), disc or drum plough to create micro-dams, and conservation tillage (CsT)

| Date | P [mm] | CN [-] | | | | | | | | | | | | | | | |
| --- | --- | --- | --- | --- | --- | --- | --- | --- | --- | --- | --- | --- | --- | --- | --- | --- | --- |
|  |  | Repetition A (slope 15–16%) | | | | | | | | Repetition B (slope 8–9%) | | | | | | | |
|  |  | CvT | | disc | | drum | | CsT | | CvT | | disc | | drum | | CsT | |
| **22 May** | 24 | 72 | | 73 | | 64 | | 64 | | 72 | | 72 | | 65 | | 65 | |
| **30 May** | 38 | 69 | | 63 | | 63 | | 62 | | 69 | | 63 | | 62 | | 62 | |
| **25 Jun** | 20 | 79 | | 75 | | 67 | | 74 | | 79 | | 75 | | 74 | | 74 | |
| **02 Jul** | 20 | 75 | | 74 | | 70 | | 74 | | 76 | | 74 | | 74 | | 73 | |
| **30 Jul** | 36 | 73 | | 73 | | 66 | | 61 | | 69 | | 68 | | 65 | | 61 | |
| **12 Aug** | 27 | 78 | | 77 | | 58 | | 67 | | 73 | | 73 | | 71 | | 68 | |
| **11 Aug** | 28 | 67 | | 66 | | 64 | | 64 | | 67 | | 64 | | 66 | | 64 | |
| **20 Sep** | 32 | 61 | | 63 | | 63 | | 62 | | 63 | | 63 | | 60 | | 63 | |
| **17 Oct** | 43 | 57 | | 56 | | 56 | | 52 | | 51 | | 53 | | 56 | | 52 | |
| **mean** |  | 70 | | 69 | | 63 | | 65 | | 69 | | 67 | | 66 | | 65 | |
| **weighted mean**  **(by P)** |  | 69 | | 68 | | 63 | | 63 | | 67 | | 66 | | 64 | | 63 | |
| **mean CvT** | 68 |  |  | |  | |  | |  |  |  | |  | |  | |  |
| **mean disc** | 67 |  |  | |  | |  | |  |  |  | |  | |  | |  |
| **mean drum plough** | 64 |  |  | |  | |  | |  |  |  | |  | |  | |  |
| **Mean CsT** | 63 |  |  | |  | |  | |  |  |  | |  | |  | |  |

### Erosion amounts

Table S4: Results of the field trials in 2018 and 2019 on the Bayer “ForwardFarm” in Huldenberg: Erosion amounts with and without the application of micro-dams (MD), with conventional tillage (CvT) or conservation tillage (CsT), respectively

| Date | P [mm] | Erosion measured [kg/ha] | | | |
| --- | --- | --- | --- | --- | --- |
|  |  | CvT | | CsT | |
|  |  | no MD | MD | no MD | MD |
| 2018 trial | | | | | |
| **02 May** | 34 | 78 | 0 | 17 | 0 |
| **15 May** | 2 | no runoff | | | |
| **24 May** | 39 | overflow, not considered | | | |
| **04 Jun** | 24 | 6.1 | 0.11 | 0.09 | 0.19 |
| **08 Jun** | 4 | no runoff | | | |
| **05 Jul** | 4 | no runoff | | | |
| **10 Aug** | 16 | 24 | 7.93 | 16 | 1.21 |
| **14 Aug** | 26 | 2193 | 1032 | 26 | 32 |
| **17 Aug** | 14 | 3.4 | 2.68 | 0.53 | 0.05 |
| **28 Aug** | 8 | no runoff | | | |
| **30 Aug** | 9 | 18.3 | 0.00 | 0.00 | 0.00 |
| **08 Sep** | 16 | 40 | 2.56 | 2.01 | 0.56 |
| **24 Sep** | 34 | 8.5 | 0.12 | 0.39 | 0.49 |
| **Sum** |  | 2371 | 1046 | 62 | 34 |
| 2019 trial | | | | | |
| **29 Apr** | 12 | no runoff | | | |
| **06 Apr** | 20 | no runoff | | | |
| **10 Apr** | 14 | no runoff | | | |
| **15 Apr** | 10 | no runoff | | | |
| **24 Apr** | 7 | no runoff | | | |
| **28 May** | 11 | 5.56 | 0.0 | 0.0 | 0 |
| **07 Jun** | 24 | 113 | 47.3 | 10.5 | 9.2 |
| **11 Jun** | 19 | 3219 | 474 | 114 | 34.7 |
| **15 Jun** | 15 | 422 | 61.6 | 57.3 | 16.0 |
| **20 Jun** | 13 | 574 | 74.7 | 56.2 | 18.2 |
| **10 Jul** | 0 | no runoff | | | |
| **15 Jul** | 4 | no runoff | | | |
| **22 Jul** | 5 | no runoff | | | |
| **29 Jul** | 48 | 218 | 78 | 33.2 | 27.6 |
| **12 Aug** | 1 | no runoff | | | |
| **13 Aug** | 2 | no runoff | | | |
| **19 Aug** | 21 | 2.22 | 1.67 | 1.0 | 1.0 |
| **03 Aug** | 11 | 134 | 57.2 | 8.2 | 0.0 |
| **12 Sep** | 8 | no runoff | | | |
| **26 Sep** | 18 | no runoff | | | |
| **01 Oct** | 45 | 830 | 222 | 131 | 69.0 |
| **04 Oct** | 25 | 1137 | 185 | 187 | 28.4 |
| **Sum** |  | 6655 | 1203 | 599 | 204 |

Table S5: Results of the field trials in 2013 on the Bayer ForwardFarm in Huldenberg: Erosion amounts for the 3 distinct management practices: conventional tillage (CvT), disc or drum plough to create micro-dams, and conservation tillage (CsT)

| Date | P [mm] | Erosion measured [kg/ha] | | | | | | | |
| --- | --- | --- | --- | --- | --- | --- | --- | --- | --- |
|  |  | Repetition A (slope 15–16%) | | | | Repetition B (slope 8–9%) | | | |
|  |  | CvT | disc | drum | CsT | CvT | disc | drum | CsT |
| **22 May** | 24 | 1.33 | 1.50 | 3.32 | 0.68 | 1.48 | 0.87 | 0.42 | 0.98 |
| **30 May** | 38 | 130 | 10.7 | 38.5 | 6.49 | 87.3 | 7.05 | 5.88 | 10.8 |
| **25 Jun** | 20 | 55.7 | 6.32 | 17.6 | 2.64 | 57.3 | 3.31 | 1.81 | 1.82 |
| **02 Jul** | 20 | 0.78 | 0.33 | 0.20 | 0.17 | 0.78 | 0.00 | 0.00 | 0.00 |
| **30 Jul** | 36 | 603 | 268 | 169 | 4.28 | 178 | 94.6 | 58.9 | 1.58 |
| **12 Aug** | 27 | 232 | 187 | 33.1 | 1.48 | 94.3 | 65.2 | 13.7 | 0.92 |
| **11 Aug** | 28 | 2.52 | 1.73 | 0.00 | 0.00 | 0.58 | 0.00 | 0.00 | 0.00 |
| **20 Sep** | 32 | 0.26 | 0.25 | 0.22 | 0.04 | 0.40 | 0.30 | 0.24 | 0.00 |
| **17 Oct** | 43 | 1.57 | 1.01 | 0.68 | 0.81 | 1.71 | 0.40 | 0.51 | 0.92 |
| **Total** | 268 | 1028 | 476 | 262 | 16.6 | 421 | 172 | 81.4 | 17.0 |

## Simulation data

### PRZM input file (.inp) - example for the trial of 2019, estimation of the MUSS C-factor (bold and underlined)

FOCUS_PRZM_SW_4.3.1, 27 Apr. 2015 PRZM 4.63 Apr. 2015

Simulation Location: Huldenberg 2019 maize

0.94 0.20 0 15.00 1 1

4

0.12 1.19 1.00 0.0054 3 9.00 18.00

1

1 0.30 99.00 90.00 3 0 0 0 0.00 250.00

1 4

1904 0708 2509 0111

**CCCE CCCG CCCM** 0.90

0.10 0.10 0.10 0.10

66 66 66 91

1

290419 070819 250919 1

Chemical Input Data:

1 1 0 0

EXSW1

11 595 0 1 4.001.0000 1.00 0.00

0. 1 0.50

Soil Series: R1

100.00 0 0 2 0 0 0 2 1 0

4300.00 .12E-07 22.70

1.0000

0.18 0.18 0.18 0.18 0.18 0.18 0.18 0.18 0.18 0.18 0.18 0.18 0.96 10.0

10.0 10.0 10.0 10.0 10.0 10.0 10.0 10.0 10.0 10.0 10.0 10.0

2.20 20.00

1 0.70 1.00

4

1 10.000 1.350 0.338 0.000 0.000 0.000

0.23105 0.23105 0.00000

0.100 0.338 0.141 1.300 0.120

10.00 11.00 15.00 0.00 0.00

2 20.000 1.350 0.338 0.000 0.000 0.000

0.23105 0.23105 0.00000

5.000 0.338 0.141 1.200 0.120

10.00 5.00 13.00 0.00 0.00

3 30.000 1.450 0.286 0.000 0.000 0.000

0.23105 0.23105 0.00000

5.000 0.286 0.111 0.300 0.030

10.00 6.00 11.00 0.00 0.00

4 40.000 1.480 0.277 0.000 0.000 0.000

0.23105 0.23105 0.00000

5.000 0.277 0.108 0.100 0.010

10.00 5.00 11.00 0.00 0.00

0

WATR YEAR 10 PEST YEAR 10 CONC YEAR 10 0

11 DAY

RUNF TSER 0 0 10.0

ESLS TSER 0 0 1.E3

PRCP TSER 0 0 10.0

IRRG TSER 0 0 1.0

SMTN TSER 0 0 1.0

SMCT TSER 0 0 1.0

SMDF TSER 0 0 1.0

INFL TSER 118 118 10.0

RFLX1 TSER 0 0 1.E7

EFLX1 TSER 0 0 1.E7

TPAP TSER 0 0 1.0
